# Agarose Spot as a Comparative Method for *in situ* Analysis of Simultaneous Chemotactic Responses to Multiple Chemokines

**DOI:** 10.1038/s41598-017-00949-4

**Published:** 2017-04-21

**Authors:** Mohaned Ahmed, Haneen A. Basheer, Jose M. Ayuso, Djevdet Ahmet, Marco Mazzini, Roshan Patel, Steven D. Shnyder, Victoria Vinader, Kamyar Afarinkia

**Affiliations:** 1 The Institute of Cancer Therapeutics, University of Bradford, West Yorkshire, BD7 1DP United Kingdom; 2 Group of Structural Mechanics and Material Modelling, Universidad Zaragoza, Zaragoza, Spain; 3 Dipartimento di Scienza e Tecnologia del Farmaco, Universitá Degli Studi di Torino, Via P. Giuria 9, 10125 Torino, Italy; 4 Department of Biomedical Engineering, Wisconsin Institutes for Medical Research, and The University of Wisconsin Carbone Cancer Center Madison, University of Wisconsin-Madison, Madison, WI 53715 USA

## Abstract

We describe a novel protocol to quantitatively and simultaneously compare the chemotactic responses of cells towards different chemokines. In this protocol, droplets of agarose gel containing different chemokines are applied onto the surface of a Petri dish, and then immersed under culture medium in which cells are suspended. As chemokine molecules diffuse away from the spot, a transient chemoattractant gradient is established across the spots. Cells expressing the corresponding cognate chemokine receptors migrate against this gradient by crawling under the agarose spots towards their centre. We show that this migration is chemokine-specific; meaning that only cells that express the cognate chemokine cell surface receptor, migrate under the spot containing its corresponding chemokine ligand. Furthermore, we show that migration under the agarose spot can be modulated by selective small molecule antagonists present in the cell culture medium.

## Introduction

Chemotactic migration of cells towards a chemoattractant source is involved in a plethora of biological processes, ranging from movement of sperm towards ovum during fertilisation^1^, to the movement of leukocytes to sites of injury during inflammatory responses^2^. Biological mechanisms which control chemotactic migration are tightly controlled in health. However, dysregulations in their functions result in enhanced or reduced chemotactic aptitude, and misdirected trafficking of cells; all of which can directly or indirectly contribute to aetiology of disease. In particular, aberration in the chemokine system, which is a major regulator of chemotaxis in pro-inflammatory and immune cells, plays a significant role in a range of inflammatory^3–10^ and autoimmune^11–17^ diseases, as well as in cancer^18–22^. In cancer, the chemokine system is associated with many different aspects of the disease and is particularly relevant in tumour cell migration and organ specific metastasis^21–25^. It has been postulated that tumour cells adopt chemokine signalling as a means of facilitating migration towards their target organs. According to this hypothesis, tumour cells acquire the expression of chemokine receptors, and thus become able to disseminate towards the organs that express their corresponding chemokine ligands^26^.

Because of the significant role that chemokines play in cancer and other diseases, chemokine receptors have emerged as attractive therapeutic targets^27–35^. A number of small molecule^30, 31^ and biologic^32^ chemokine receptor antagonists have been described as therapeutic agents, supported by a number of *in vitro* methods to assess their potency against chemotactic responses^33–35^. However, these drug discovery efforts are hampered by the lack of methods to rigorously interrogate the complex pharmacology in the chemokine system.

The chemokine system comprises of 49 chemokine ligands divided into four subfamilies (CXCL1–18, CCL1-CCL28, XCL1-2, and CX_3_CL1) which bind and activate 18 chemokine cell surface receptors (CXCR1-6, CCR1-10, XCR1 and CX3CR1), and also bind four atypical receptors (ACKR1-4)^36–38^. Although there are chemokine ligands and receptors which are uniquely paired within each subfamily, generally there is significant promiscuity between ligands and receptors. This means that some chemokine receptors can be activated by more than one chemokine ligand, and that some chemokines can bind and activate more than one chemokine receptor. For example, chemokine receptor CXCR4, which is one of the most studied chemokine receptors, is selectively activated by chemokine CXCL12, whereas chemokine receptor CCR7 is activated by two chemokines, CCL19 and CCL21. Also, whilst chemokine CCL20 exclusively activates chemokine receptor CCR6, chemokine CCL5 activates receptors CCR1, CCR3 and CCR5.

Moreover, it is becoming clear that often more than one chemokine axis contributes to the progression of certain diseases^39–43^. Therefore, there are now a number of examples where antagonism of more than one chemokine receptor is considered advantageous as a treatment strategy^44–47^. For example, there is some clinical evidence to support the hypothesis that the CXCR4 and CCR7 axes may work in tandem to promote the dissemination of cancer. Co-expression of CXCR4 and CCR7 in breast^48^, cervical^49^, thyroid^50^, and gastric^51^ correlates to poorer prognosis and exacerbated metastasis, compared to expression of either receptor alone.

For both these reasons, development of *in vitro* models that enable a direct comparative assessment of the simultaneous chemotactic response of cells towards different chemokines would be highly useful for understanding the functional role of each axis in cell migration, and allow direct comparison of the relative efficacies of different antagonists.

Here, we report a simple-to-perform experimental reconfiguration of the under-agarose method^52–54^, which permits the time-dependant analysis of cell migration in response to multiple chemokines. Analysis of the number of cells and the distance they travel under the spot provides quantitative information on the chemotactic aptitude of cells towards different chemokines. The method is known as the “agarose spot” assay^54–57^. Whilst there are a number of methods to study cell migration and chemotaxis in response to a single chemoattractant, such as for instance the Boyden chamber^58^, Zigmond chamber^59^, Dunn chamber^60^, and Insall chamber^61^, none of these is adaptable for the investigation of simultaneous cellular migration in response to multiple chemoattractants. The agarose spot assay has obvious practical advantages over these methods, as it is more convenient and less time consuming to run one assay for multiple chemokines, than multiple assays for each individual chemokine. In addition, using this configuration of the agarose spot assay, direct comparisons can be drawn more confidently between chemotactic aptitudes towards different chemokines, because all experiments are carried out simultaneously and under uniform conditions. Furthermore, it provides an opportunity to assess if combined antagonism of multiple chemotactic axes can be additive (see later).

To exemplify the use of this protocol, and in view of above mentioned evidence suggesting that the CXCR4 and CCR7 axes may work in tandem to promote the dissemination of cancer^48–51^, we report its application to CXCL12 (ligand for CXCR4), and CCL19 and CCL21 (ligands for CCR7) triggered cell migration, and the effects of small molecule antagonists of these receptors on the migration of cells.

## Results

### Setup of an agarose spot assay

**Figure 1:**
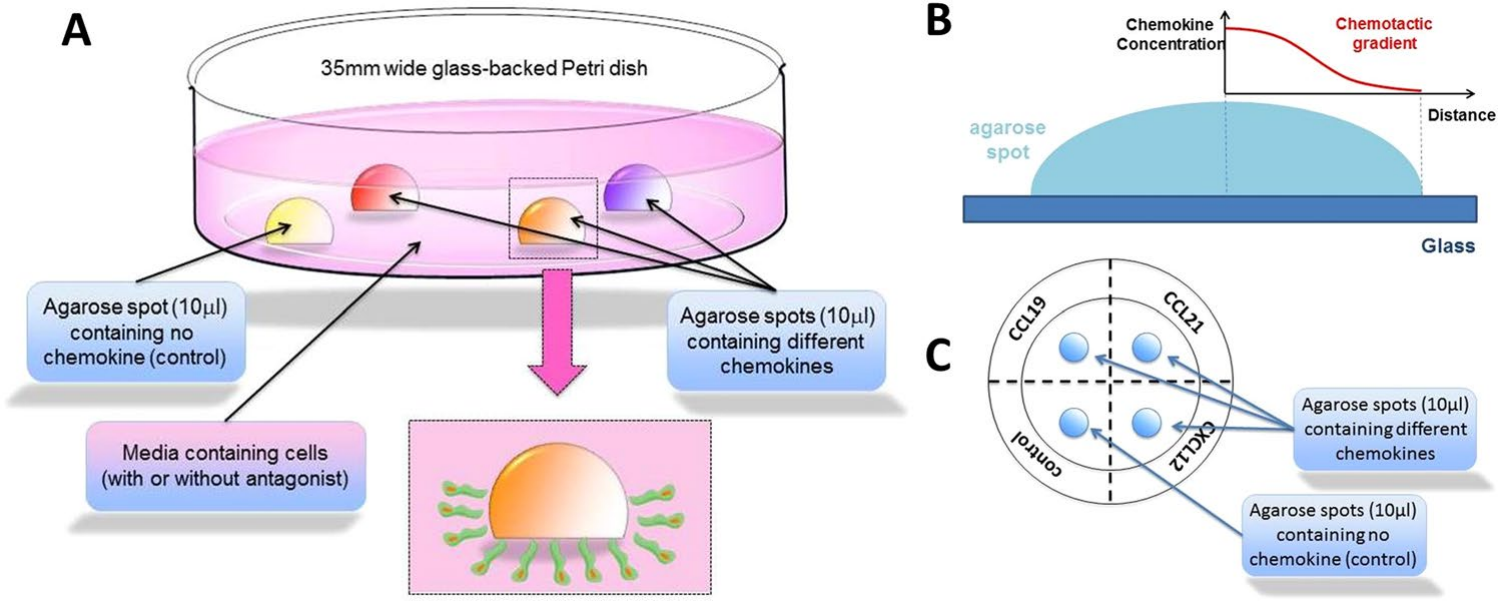
(**A**) A schematic representation of a glass backed 35 mm petri dish with three agarose drops containing chemokines, and one agarose drop containing PBS as control. Cells migrate under the agarose spot in response to the chemokines. (**B**) A schematic representation of chemotactic gradient established within the agarose spot (**C**) A schematic representation of glass back Petri dish with agarose drops from the top, dotted line and writing on the bottom of the Petri dish are also shown.

In the agarose spot assay, chemokine molecules are initially contained in an agarose drop which is applied to a glass bottom Petri dish as a spot, and immersed under media containing cells (Fig. 1A). It should be noted that even though some chemokines possess glucosamine aminoglycan (GAG) binding domains at their N-terminus^36^, it has been previously shown that the rate of diffusion in agarose is not influenced by the presence of these domains^62^. The negative control is an agarose drop containing no chemoattractant. Over time, chemokine molecules slowly diffuse outwards from the peripheral regions of the agarose spot, creating a transient chemotactic gradient within the agarose spot (Fig. 1B)^63^. Cells expressing the corresponding cognate chemokine receptors, and only those cells, respond to the chemokine and migrate against this gradient by moving into the space between the agarose and the glass, towards the centre of the drop. We found that the analysis of the number of cells under the spot provides a very convenient and quantitative measure of the chemotactic aptitude. In addition, average distance travelled by cells, velocity of cell movement or the surface area under the agarose spot covered by migrating cells can be recorded over time as a video (or concatenated images captured at specific time intervals) which can also be analysed to measure chemotactic aptitude (see Supplementary Information S1 as an example). Furthermore, the media can be supplemented by small molecule antagonists, antibodies or other agents, and thus we can assess their effect on modulating the migratory response (see below)^55^. *A video tutorial showing the procedure for setting up the agarose assay is provided* (Supplementary Information S2).

**Figure 2:**
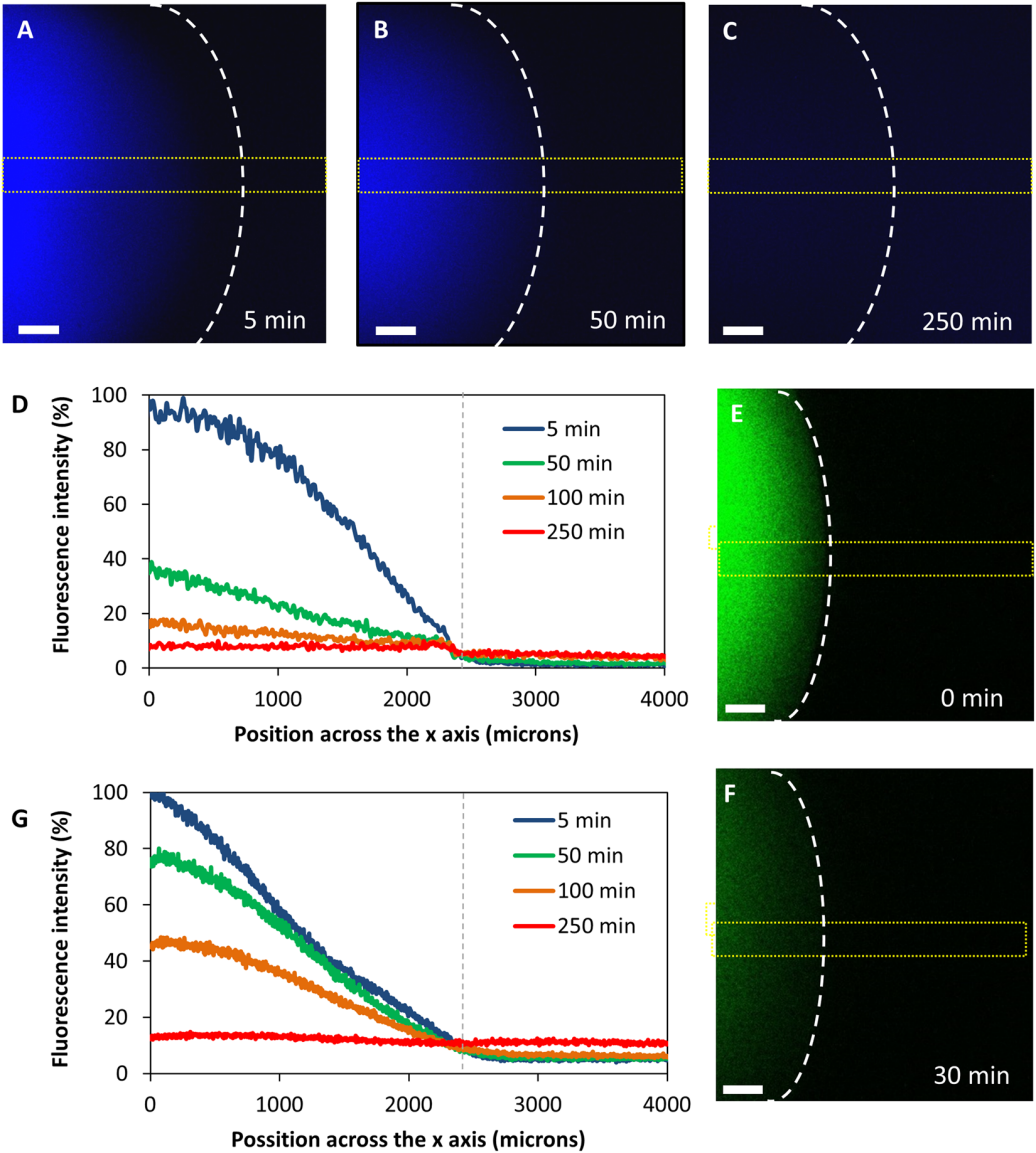
(**A–C**) Diffusion of Cascade Blue dextran (mean MW = 10 kDa) from an agarose spot at different time intervals time. The edge of the spot is shown by dotted white line. (**D**) Fluorescence intensity along a central strip of the agarose spot (shown as a dotted yellow line) at different time intervals. The edge of the spot is shown by dotted grey line. (**E** and **F**) Diffusion of Fluorescein dextran (mean MW = 40 kDa) from an agarose spot at 0 and 30 minutes time intervals. (**G**) Fluorescence intensity along a central strip of the agarose spot at different time intervals. The edge of the spot is shown by dotted grey line.

To demonstrate that a gradient is established, we investigated the diffusion behaviour of Cascade Blue fluorescent dextran (mean MW = 10 kDa, which is close to that of most chemokines) and FITC labelled dextran (mean MW = 40 kDa). Thus, we carried out an agarose spot experiment using fluorescent dextran loaded in the agarose spot. We then recorded the fluorescence intensity along a central strip of the agarose spot at four time intervals (Fig. 2A–C and E,F). As can be seen, a concentration gradient is established across the agarose spot, as evidenced from the drop in fluorescence in both cases. Furthermore, that concentration gradient depends on MW of the dextran (Fig. 2D and G and see Discussions).

### Migration under the agarose spot corresponds to the expression of chemokine receptor

We had previously validated the agarose spot assay by showing that MDA-MB-231 cells, which we showed express CXCR4, migrate under an agarose spot containing CXCL12 and that the migration can be modulated by addition of a selective monoclonal antibody antagonist of CXCR4 in the medium^55^. In order to compare the chemotactic aptitude of other cell lines towards multiple chemokines, we first required cell lines with contrasting expression of the chemokine receptors CXCR4 and CCR7, and responsiveness to their cognate ligands. We screened cells from our cell bank for the expression of chemokine receptors CCR7 and CXCR4 and found that PC-3 (human prostate cancer) cells express high levels of both CXCR4 and CCR7 (Supplementary Information S3). However, whilst SW480 (human colon cancer) cells do express CXCR4, their expression of CCR7 is negligible (Supplementary Information S3). Furthermore, we confirmed that the receptors on PC-3 and SW480 cells are functional and that the cells selectively respond to the chemokines for which they express receptors. In both intracellular calcium mobilisation (calcium flux) assay (Supplementary Information S4) and scratch assay (Supplementary Information S5), PC-3 cells respond to CCL21, CCL19 and CXCL12, respective ligands for CCR7 and CXCR4, whilst the SW480 cells similarly respond to CXCL12, but not to CCL19 or CCL21.

**Figure 3:**
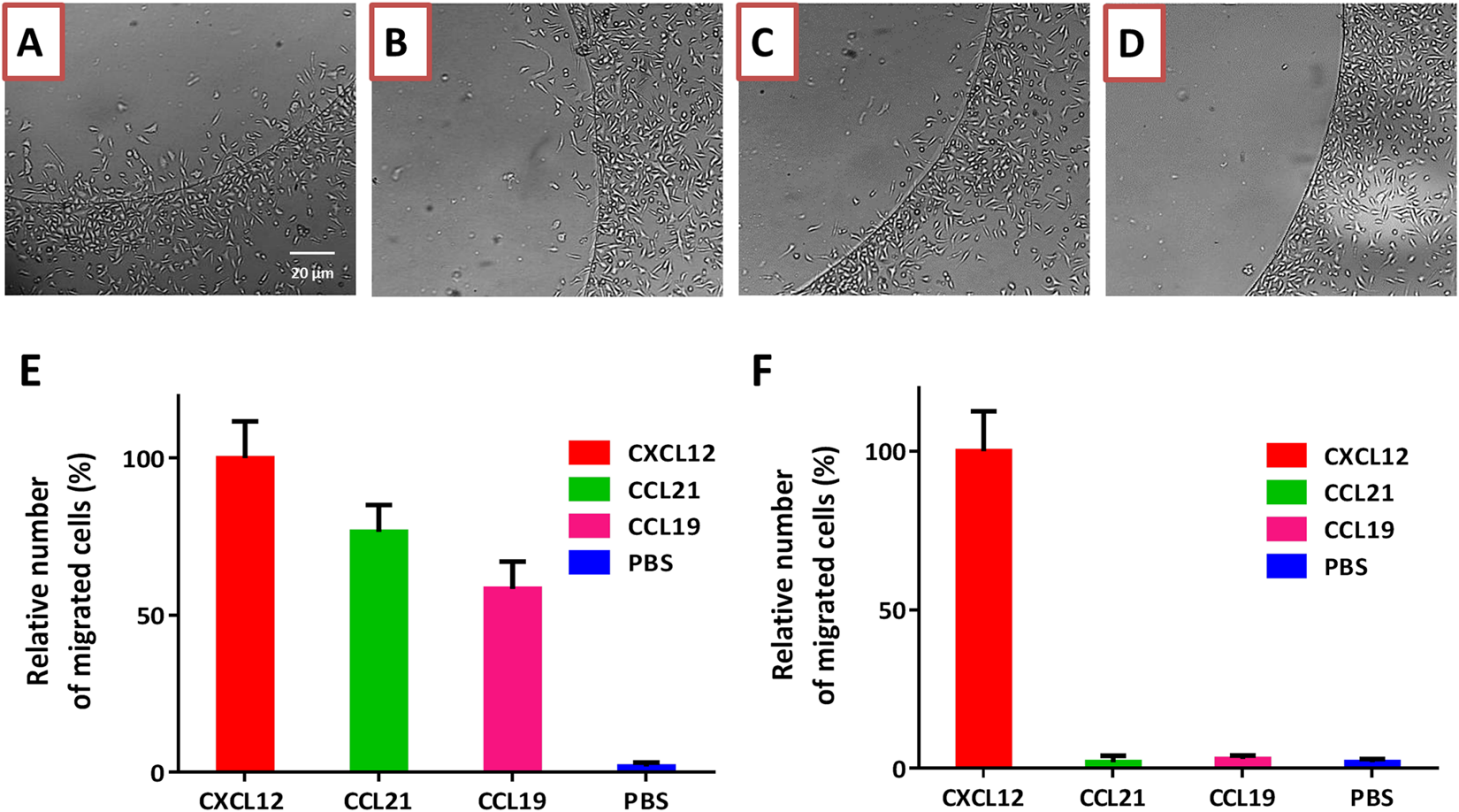
Cells migrate under the agarose spot containing a chemokine according to the expression of the cognate chemokine receptor. PC-3 cells which express chemokine receptors CXCR4 and CCR7 migrate under agarose spot containing ligand for CXCR4, CXCL12, (**A**), and ligands for CCR7, CCL21, (**B**) and CCL19, (**C**), but do not migrate under agarose spot containing no chemokine, (**D**). (**E**) Relative chemotactic aptitude of PC-3 cells under agarose spot containing 100 nM CXCL12 (ligand for CXCR4), and agarose spot containing 100 nM CCL19 and 100 nM CCL21 (ligands for CCR7), but not under agarose spot containing no chemokine (n = 3). (**F**) SW480 cells which express chemokine receptor CXCR4 but not CCR7, migrate under agarose spot containing 200 nM CXCL12 (ligand for CXCR4), but not under agarose spot containing 200 nM CCL19 and 200 nM CCL21 (ligands for CCR7), or agarose spot containing no chemokine (n = 3).

Using PC-3 cells, we carried out an experiment with four agarose spots on the same 35-mm dish, with one as a control containing no chemoattractant, and the other three containing CXCL12, CCL19 and CCL21. We observed that the PC-3 cells do migrate under the agarose spots containing chemokines CXCL12, CCL19 and CCL21 (Fig. 3A–C), but do not migrate under the agarose spots containing no chemoattractant (Fig. 3D).

In contrast to PC-3 cells, SW480 cells do not significantly express CCR7, but do express CXCR4. Therefore, we repeated this experiment and allowed SW480 cells to migrate under CXCL12, CCL19 and CCL21 spots. In this experiment, we observed migration under the CXCL12 spot but not under CCL19 nor CCL21 spots (even at 200 nM concentration) (Fig. 3F).

### Migration under the agarose spot is directional and chemotactic

As indicated above, a number of criteria can be used to analyse and quantitatively compare the chemotactic response of cells under the agarose spots. Most convenient method is to count the number of cells under each spot, or measure the surface area under the agarose spot covered by migrating cells, over a time interval.

**Figure 4:**
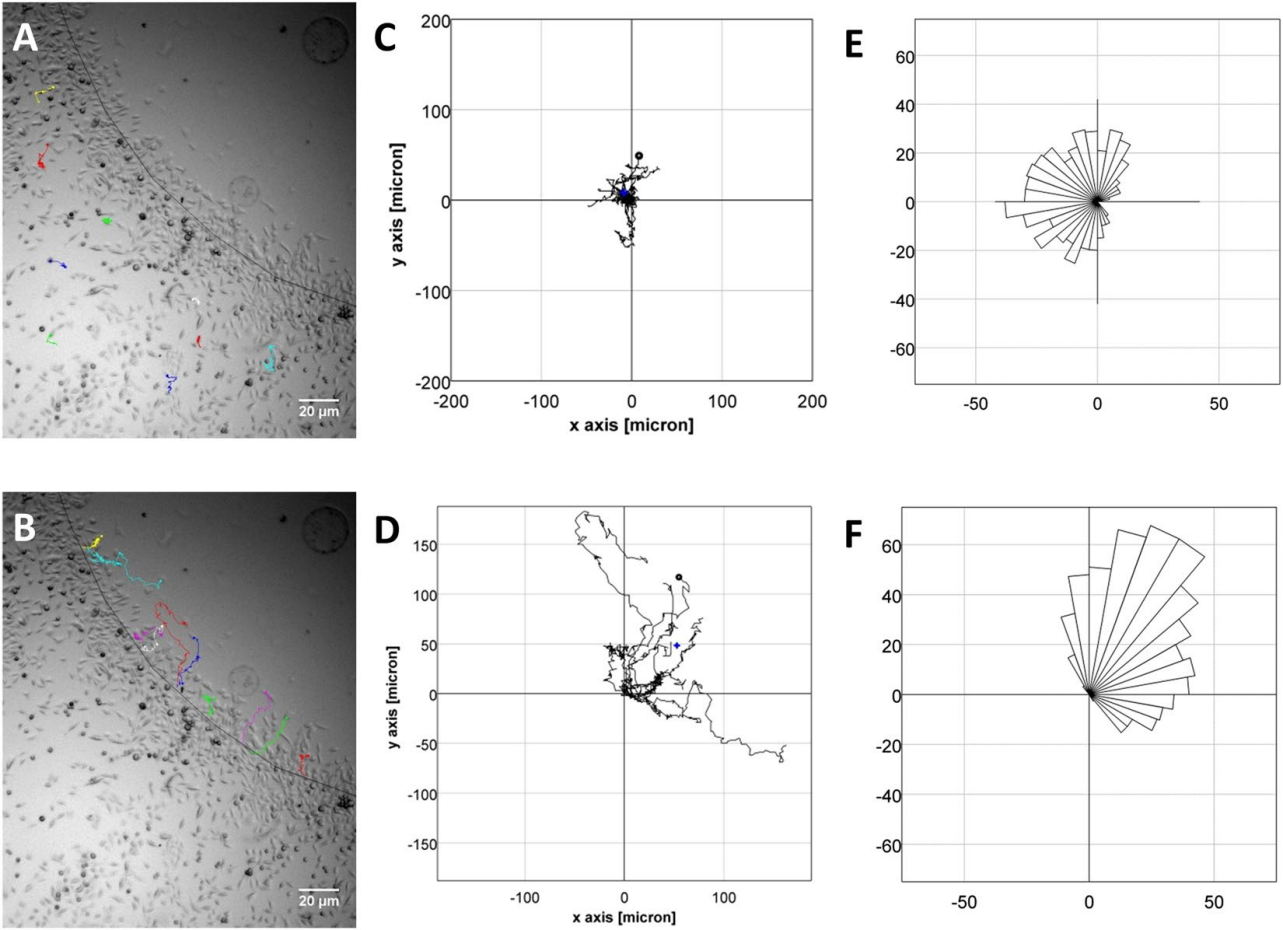
(**A**) Tracks of ten randomly selected PC-3 cells away from the agarose spot containing CCL21. (**B**) Tracks of ten randomly selected PC-3 cells from the edge of the agarose spot containing CCL21. (**C**) Plots of overlapped tracks for PC-3 cells away from the agarose. Center of mass is (−3.4 µm, 13 µm), mean of accumulated distance travelled is 218 ± 82 µm, average velocity is 0.13 µm/min. (**D**) Plots of overlapped tracks for PC-3 cells from the edge of the agarose. Center of mass is (53 µm, 48 µm), mean of accumulated distance travelled is 320 ± 84 µm, average velocity is 0.30 µm/min. (**E**) Rose diagram showing lack of directionality in PC-3 cells away from the agarose spot. (**F**) Rose diagram showing directionality towards the center of the spot in PC-3 cells under the agarose spot.

In addition, average distance travelled by cells, velocity of cell movement or the surface area under the agarose spot covered by migrating cells can be recorded over time as a video (or concatenated images captured over a time period) which can then be analysed to measure chemotactic aptitude. For example, we used concatenated images, taken every 5 minutes, of an experiment with PC-3 cells with an agarose spot containing CCL21 (Supplementary Information S1). Ten cells, randomly selected from the edge of the spot and ten cells randomly selected away from the spot were tracked and analysed (Fig. 4). The analysis of the cell movements shows that the cells under the spot move a longer distance, move more quickly than those cells which are away from the spot. Furthermore, cells which are under the spot move towards the center of the spot, whereas those away from it move non-directionally. To visualize cell orientation independently of cell speed, data were plotted as Rose diagrams which indicate the distribution of migration angles. (Fig. 4E and F).

### Migration under the agarose spot is selectively modulated by small molecule antagonists

Having shown that migration under the agarose spot corresponds to expression of chemokine receptors, we then set out to investigate the selective antagonism of individual receptors in this experiment.

**Figure 5:**
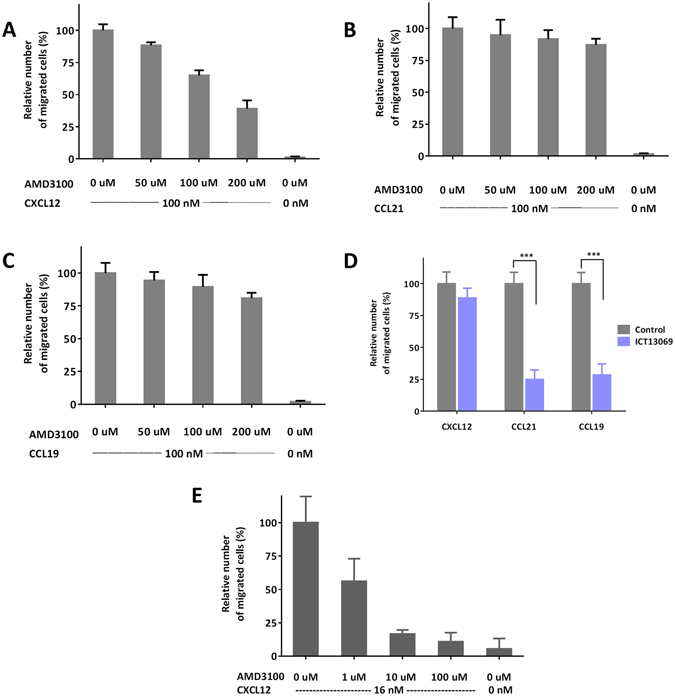
Cells migration under the agarose spot can be selectively modulated by antagonists. (**A**) Migration of PC-3 cells under agarose spot containing 100 nM CXCL12 (ligand for CXCR4), is modulated in a dose-dependent manner by CXCR4 selective antagonist, AMD3100. (**B** and **C**) However, migration of PC-3 cells under agarose spot is not abrogated by CXCR4 selective antagonist, AMD3100 for the spots containing 100 nM CCL21 and 100 nM CCL19 (ligands for CCR7). (**D**) Migration of PC-3 cells under agarose spot containing 100 nM chemokines in the absence (grey bars) or presence (purple bars) of CCR7 antagonist ICT13069 (10 µM) shows that migration against CXCL12 is not modulated but that migration is reduced against CCL19 and CCL21. (**E**) Comparison of the inhibition of CXCL12 induced migration of PC-3 cells by AMD3100 in a Boyden assay.

AMD3100 is a selective CXCR4 antagonist previously shown not to antagonise other chemokine receptors including CCR7^64, 65^. To demonstrate the application of the agarose spot assay to assess selectivity of small molecule antagonists, PC-3 cells were allowed to migrate under CXCL12, CCL19 and CCL21 spots in the same Petri dish, in the presence of a range of concentrations of CXCR4 antagonist AMD3100 in the media. We observed a dose dependant reduction in PC-3 cell migration under the CXCL12 agarose spot (Fig. 5A). However, no significant reduction of migration was observed under the CCL19 and CCL21 spots (Fig. 5B and C). This observation is consistent with our expectations, since AMD3100 antagonises the migration against CXCL12, but it does not interfere with the migration against CCL19 or CCL21, as it does not antagonise CCR7, the corresponding receptor for these chemokines.

In another experiment, PC-3 cells were allowed to migrate under CXCL12, CCL19 and CCL21 spots in the same Petri dish, in the presence or absence of CCR7 small molecule selective antagonist ICT13069 in the media. In the presence of 10 µM ICT13069, migration of PC-3 cells under the CCL19 and CCL21 spots was significantly reduced (Fig. 5D). However, no significant reduction of migration was observed under the CXCL12 containing spot. This observation is again consistent with our expectations, since a CCR7 antagonist with selectivity over CXCR4, antagonises the migration against CCL19 or CCL21, but it does not interfere with the migration against CXCL12.

Interestingly, we observed a small reduction in responsiveness to CCL19/CCL21 in the presence of the selective CXCR4 antagonist, and similarly, a small reduction in responsiveness to CXCL12 in the presence of the selective CCR7 antagonist.

Finally, whilst the agarose spot method has the advantage of enabling simultaneous measurement towards multiple chemoattractants, we still wished to draw a comparison between this and one of the established methods for measuring chemotactic aptitude. So, we compared dose-dependent inhibition of the migration of PC-3 cells using chemokine antagonist AMD3100 in the agarose spot and a two-chamber Boyden assay (Fig. 5E). Boyden chamber is one of the most common means of assessing chemotactic aptitude and is widely used to assess migration of cells through a membranous barrier and its inhibition by small molecule antagonists. To make a comparison, we correlated the relative number of cells migrated under the agarose spot with the relative number of cells migrated through a 6.5 mm thick polycarbonate membrane with 8.0 µm pore size (n = 3 in each case). We note that whilst the initial concentration of the chemokine in the agarose spot is 100 nM, the concentration experienced by cells migrating under agarose spot edge is more similar to the concentration experienced by cells migrating in a Boyden assay (16 nM) as shown from computational studies^63^ and also in Fig. 2. The Boyden assay does have a shorter incubation time, but we found that there was less variation in relative number of cells migrated under the agarose spot leading to smaller error bars in thee agarose spot assay.

## Discussion

Chemokine-induced migration of cells is associated with the chemoattractant’s concentration gradient. Therefore, we wanted to demonstrate that such a concentration gradient exists under the agarose spot. We reasoned that once the agarose spot containing a chemokine is immersed in medium, the chemokine peptide begins to leach out, but does so more efficiently from the regions in the periphery of the spot. It would be expected therefore that after a period of time, regions close to the interface between the agarose spot and the medium will have a lower concentration of chemokine compared to regions further inside the spot, hence creating a transient concentration gradient. To demonstrate this was the case, we investigated the diffusion behaviour of Cascade Blue fluorescent dextran (mean MW = 10 kDa) as a surrogate for chemokines. The rate of diffusion of chemokines correlates with their molecular weight^66–68^. Therefore, we would expect that the rate of diffusion of this dextran would be comparable to that of most chemokines such as CXCL12 (MW = 8.0 kDa), CCL19 (MW = 8.8 kDa) and CCL21 (MW = 12.2 kDa).

As can be seen (Fig. 2A–C), a concentration gradient is established across the agarose spot as evidenced from the drop in fluorescence over distance from the centre of the spot. This concertation gradient however is transient and weakens until it eventually disappears over time.

Interestingly, the diffusion of CXCL12 from the agarose spot in this experiment was previously modelled computationally by Szatmary^63^. Reassuringly, the experimental rate of diffusion of Cascade Blue dextran, which has a similar MW to that of CXCL12, is comparable to that calculated in that investigation, confirming Szatmary’s to be an appropriate model for the rate of chemokine diffusion from the agarose spot.

It should also be noted that the rate of diffusion of macromolecules in agarose is inversely proportional to the square root of their molecular weight. Since the molecular weight of the majority of chemokines is within a relatively narrow range of 8–12 kDa, this observation gave us confidence that the rate of diffusion of different chemokines, and hence the concentration gradients from different chemokines would be relatively similar. Hence we can expect that any differences in cell migration under different spots containing different chemokines would not be due to differences in concentration gradient, provided the initial concentration of chemokines in different agarose spots are similar. Indeed, the concentration gradient observed in the agarose spot containing FITC labelled dextran (mean MW = 40 kDa) was approximately half that of Cascade Blue dextran (mean MW = 10 kDa) as expected (Fig. 2E–G).

The experimental configuration of agarose spots enables the study of migration of cells towards multiple chemokines simultaneously and under the same conditions. Here, we applied the assay to determine relative chemotactic responses of different cell lines. In the example of SW480 cells, we observed migration under CXCL12 agarose spot but not under CCL19 or CCL21 agarose spots. This observation is wholly expected, and consistent with the observed lack of significant expression of CCR7 in SW480 cells. On the other hand, PC-3 cells express both CCR7 and CXCR4, however their chemotactic response toward CCL19 and CCL21, is relatively lower than that towards CXCL12 (Fig. 3E). To compare our observations with other functional properties, we measured intramolecular calcium ion mobilisation and looked at cell motility in scratch assays. We measured the intramolecular calcium ion mobilisation in PC-3 cells treated with the same concentration of chemokines CXCL12, CCL19 and CCL21. Activation of chemokine receptors is known to cause release of calcium ions in the cytoplasm as a prelude to cytoskeletal changes that enable cell migration. In a calcium mobilisation (flux) assay, the increased concentration of cytoplasmic calcium is measured by addition of a calcium-dependent fluorescent dye. Interestingly, relative increase in concentration of calcium ions in the cytoplasm (as measured by transient increase in fluorescence) appears to correlate with the relative chemotactic aptitude towards the three chemokines (Supplementary Information S3). Similarly, we measured increased cell motility in scratch assays with PC-3 and SW480 cells in the presence of CXCL12, CCL19 and CCL21, compared with control (no chemoattractant). We similarly observed increases in gap closure in PC-3 cells after addition of CXCL12, CCL19 and CCL21 commensurate with the expression and functional ability of the corresponding receptors. However, gap closure in SW480 cells was only observed in the addition of CXCL12 confirming that the CXCR4 receptors in the cells are functionally active (Supplementary Information S5).

Correlation between the results of these other two assays with that of agarose spot assay gives us confidence in the robustness of the agarose spot assay as a means of assessing the relative potency of chemokine agonists.

Another useful application of the agarose spot assay is in determining functional selectivity of small molecule antagonists against multiple chemokine receptors simultaneously and in the same experiment. In addition to the convenience and time-saving it provides, the agarose spot assay gives an unparalleled opportunity to ensure a more reliable comparison of antagonism of different chemokine receptors by the same chemical agent.

AMD3100 is reported as a selective CXCR4 antagonist with no modulation of other chemokine receptors, including CCR7^64^. When AMD3100 is added to the medium, migration under the agarose spot containing CXCL12 is inhibited in a dose dependent manner, which confirms its potency as a CXCR4 antagonist. ICT13069 is a small molecule CCR7 antagonist discovered in our laboratory and, when added to the medium, inhibits migration of cells under agarose spots containing CCL21 and CCL19. Therefore, the agarose spot is clearly a useful technique to assess the potency of chemokine antagonists. Interestingly, comparison between a Boyden and the agarose spot assays, shows that they both provide reliable means of assessing dose-dependent inhibition of cell migration. Whilst the the Boyden assay does have a shorter incubation time, the agarose spot assay has the advantage of allowing assessment against more than one chemokines.

Since AMD3100 has no CCR7 antagonism, we would have expected no change in migration of cells under agarose spots containing CCL21 and CCL19. In fact we observed a very small, but observable drop in migration of cells under those spots. Similarly, we observed a small drop in migration of cells under the CXCL12 containing agarose spot upon treatment with CCR7 antagonist. Obviously, both observations can be attributed to a lack of target selectivity. However, in view of the well-established selectivity of AMD3100 for the CXCR4 receptor, that seems unlikely. This observation merits further investigation; however, it is the first direct observation that antagonism of one chemotactic receptor, can functionally influence the responsiveness of other chemotactic receptors.

## Conclusions

The protocol described herein provides a simple and practical approach to simultaneous measurement of chemotactic response of cells towards multiple chemoattractants, such as chemokines. In addition, we have also shown that inhibition of migration against different chemokines by small molecule antagonists can be quantitatively compared using this protocol. Overall, the agarose spot assay provides an opportunity to study aspects of chemotactic migration and its inhibition, which are not available using other established techniques.

## Materials and Methods

### Chemicals and biologicals

Recombinant CXCL12 (catalogue number 350-NS), CCL19 (catalogue number 361-MI-025), CCL21 (catalogue number 457-6C-025) and anti-human CXCR4 monoclonal antibody (catalogue number MAB173) were purchased from R&D Systems (Abingdon, OX14 3NB, UK). Alexa Fluor 488 anti-human CCR7 antibody (catalogue number 353206) and the recommended isotype control (catalogue number 400233) were purchased from Biolegend (London, NW5 1LB, UK). Ultrapure^TM^ low-melting agarose (catalogue number 16520–050) was purchased from Invitrogen (Paisley, PA4 9RF, UK). CXCR4 antagonist AMD3100^65^ (catalogue number 3299), was purchased from Tocris Biosciences (Missouri 63021, USA). CCR7 Antagonist ICT13069 was synthesised at the Institute of Cancer Therapeutics (Bradford, BD7 1DP, UK). Cascade Blue-10 kDa (Catalogue number D1976) and Fluorescein-40 kDa (Catalogue number D1845) dextrans were purchased from ThermoFisher (Loughborough, LE11 5RG, UK). Vectashield hardset antifade mounting medium with DAPI (catalogue number H-1500) was purchased from Vector laboratories, (Peterborough, PE2 6XS, UK). Bovine serum albumin (catalogue number A2153) was purchased from Sigma-Aldrich (Gillingham, SP8 4XT, UK).

### Cells and cell culture

Human prostate PC-3 and colorectal SW-480 adenocarcinoma cell lines were obtained from the ATCC (Middlesex, TW11 0LY, UK). The cells were cultured as monolayers at 37 °C and 5% CO_2_ atmosphere in RPMI1640 medium (Sigma-Aldrich, catalogue number R5886), supplemented with 10% foetal bovine serum (FBS), 1 mM sodium pyruvate (sigma-Aldrich, catalogue number, S8636) and 2 mM L-glutamine (sigma-Aldrich, catalogue number G7513).

### Equipment

Disposable glass-bottomed plastic 35 mm Petri dishes with lid (catalogue number P35G-1.5-20-C) were purchased from MatTek Corporation (Ashland, MA, USA). These dishes have a usable glass surface area with a 20 mm diameter. For Boyden assays, we used 6.5 mm Transwell inserts with 8.0 µm pore polycarbonate membrane (catalogue number 3422) from Corning (Flintshire, CH5 3XD, UK). Flow cytometry was performed on a BD FACSCalibur instrument and the results were analysed by BD CellQuest Pro software version 5.1.1. Images were recorded using LumaScope 500 (Etaluma Inc., Carlsbad, CA 92010, USA) with 20x magnification. Cell migration was imaged by timelapse microscopy using a Nikon Eclipse TI confocal microscope (40x magnification) installed within an enclosed cabinet maintained at 37 °C and 5% humidified CO_2_ atmosphere. Photographs were taken at 5 minute intervals. The data were exported as multipage TIFF files to enable manual cell tracking using the ImageJ ‘Manual Tracking’ plug-in. The ImageJ ‘Chemotaxis and Migration Tool’ plug-in was used to quantitate data (see the section on analysis).

### Methods

100 mg of low–melting point agarose (catalogue number 16520–050, Thermo﻿ Fisher Scientific﻿) was placed into a 100-mL beaker and diluted into 20 mL PBS to make a 0.5% agarose solution. The mixture was heated on a hot plate, and stirred to facilitate complete dissolution. Once all agarose particles were dissolved, the beaker was taken off the heat and cooled down to 40 °C.

Agarose solution of CXCL12 was prepared as follows: 10 µg lyophilized CXCL12 was reconstituted by adding 100 μL of sterile phosphate-buffered-saline solution (PBS) containing 0.1% BSA to afford a 12.5 μM stock solution. To prepare a 100 nM CXCL12/agarose solution, 1.6 μL of this CXCL12 stock solution was mixed with 18.4 μL of PBS. The resulting 20 μL was then mixed with 180 μL of 0.5% agarose solution at 40 °C. Agarose solutions of other chemokines were similarly prepared from the corresponding stock solutions by adjusting the second PBS dilution as required. To prepare the control agarose solution, 20 μL of PBS was mixed with 180 μL of 0.5% agarose solution at 40 °C.

Using a marker pen, a cross was drawn on the back of the plate to form four quadrants, one for each of the four spots (Fig. 1C). The ends of 200 μL pipette tips (catalogue number 70.760.012; Sarstedt, Leicester, LE4 1AW, UK) were cut with scissors by about 2 mm in order to facilitate the transfer of the viscous agarose solution and the formation of the spot. Using these cut pipette tips, 10 μL drops of agarose solution (either control containing PBS, or containing chemokine) were applied onto each of the quadrants of the Petri dish as shown (Fig. 1C). Depending on the purpose of the experiment, spots containing the same chemokine or different chemokines (to allow comparison) can be applied for each dish. If required, the spots can be identified by writing on the backside of the glass. The Petri dish containing the spots was then cooled for 5 minutes in a 4 °C fridge to allow the agarose spot to set.

1 mL of cell suspension in 10% FBS cell culture medium, either containing the antagonist(s), or control (no reagent), was plated into the dishes which were then incubated at 37 °C to allow the cells to adhere. We recommend a cell density of about 6.0 × 10^4^ cells/mL as a starting point, but this can be adjusted. In the experiments described here, 6.0 × 10^4^ cells/mL of PC-3 and 6.5 × 10^4^ cells/mL of SW480 were used. After 4 hours, the culture media was replaced with 0.1% FBS, either containing the antagonists or no reagent (as control), and the dish was returned to the 37 °C incubator. After 16 h, the agarose spots were analysed by counting the total number of invading cells using a microscope. The purpose of the media change is to ensure no cell proliferation during the overnight incubation period.

#### Flow cytometry

Cells were used at 60–70% confluency. Around half million cells were suspended in 100 µl of 4% aqueous paraformaldehyde for 10 min at room temperature, then centrifuged, thoroughly washed twice and re-suspended in 1% bovine serum albumin (BSA). To determine the expression of CCR7, cells were incubated with conjugated anti CCR7 (2:100) for 30 min, then washed 3 times with 1% BSA, re-suspended in 500 µl of 1%BSA and analysed. The isotype matched control for CCR7 (2:100) was similarly used. To determine the expressions of CXCR4, cells were incubated with anti CXCR4 (5:100) for 1 hour and then washed 3 times with 1% BSA. After addition of the secondary antibody conjugated to R-phycoerythrin, cells were incubated for one hour on ice, then washed 3 times with 1% BSA, re-suspended in 500 µl of 1% BSA and analysed. The change in fluorescence intensity was calculated by comparing the fluorescence intensity mean of stained cells relative to their isotype control for each cell line.

#### Scratch assay

350 µL of a suspension of PC-3 cells in serum free RPMI media (8.5 × 10^5^ cells/mL) was added to each well of a 24 well flat-bottom plate. The plate was incubated at 37 °C overnight to ensure the formation of a uniform monolayer. Using the narrow end of a P200 pipette tip, a wound was scratched across each well. The medium and cell debris were removed, and the wells were gently washed with 350 µL of PBS. PBS was removed and replaced by 350 µL of either the controls (0% FBS in RPMI medium or 0% FBS in RPMI medium containing a chemokine) or treatment (0% FBS in RPMI medium containing AMD3100 or ICT13069 or 0% FBS in RPMI medium containing AMD3100 or ICT13069 with the respective chemokine). Images were obtained from plates at 0 h and 14 h and were analysed by ImageJ^68^. The experiment was similarly repeated with SW480 cells (8.5 × 10^5^ cells/mL) but using 2% FBS RPMI media throughout and analysing the plates after 21 h.

#### Transwell (Boyden) assay

PC-3 cells were cultured in serum-free medium for 24 hours prior to the experiment. 150 µl of cell suspension (6.7 × 10^5^ cells/mL) was added to the upper chamber of a 6.6 mm diameter, 8.0 µm pore size Transwell filter. For controls, 600 µl serum-free medium was added to the lower chamber, for CXCR4 mediated chemotaxis, 600 µl serum-free medium supplemented with CXCL12 (16 nM) was added to the lower chamber. For the CXCR4 antagonist experiment, cells were pre-incubated for 1 hr with AMD3100 at various concentrations (100 µM, 10 µM, and 1 µM) and loaded to the upper chambers. The chambers were incubated for 3 hours at 37 °C under 5% CO_2_ atmosphere.

The upper face of the Transwell filter was cleaned with a cotton swab. The migrated cells to the lower face of the filter were fixed by immersing them in 70% aqueous ethanol for 2 minutes at room temperature. The filters were air dried, cut and mounted onto microscope slides using DAPI containing mounting medium for nucleus staining and left to dry for 1 hour at 4 °C in the dark and analysed by fluorescence microscope (Leica DM2000; Leica Microsystems, UK). The cell numbers in various fields were counted for each chamber and averaged. Data is presented as the mean ± SD (standard deviation) of at least 3 independent experiments.

#### Calcium mobilisation

Cells were seeded into each well of a 0.1% gelatine-coated 96-well black-wall microtiter plate at a density of 1.0 × 10^5^ cells/well. After 24 h, the growth medium was replaced with 100 μL of the dye loading solution (Molecular Probes™ Fluo-4 NW, Invitrogen catalogue number F36206, Thermo Fisher Scientific). The plates were incubated at 37 °C for 1 hour. 20 μL of a given concentration of the antagonist in assay buffer, or assay buffer as control, was added to each well and the plate was incubated at 37 °C for an additional 30 minutes. The plate was transferred into a Fluoroskan Ascent FL instrument (ThermoScientific) and the fluorescence change in response to the addition of 20 μL chemokine (to give 100 or 200 nM in-well concentration in assay buffer) was measured at 37 °C temperature (Ex 485 nm, Em 538 nm). IC_50_ is calculated as the concentration of the antagonist required to half the maximum fluorescence change in response to CXCL12. Data is presented as the mean ± SD of at least 3 independent experiments.

### Analysis

The agarose spots were observed under a microscope (x40 magnification). The edges of the spots are discernible and the cells migrating under the spot are clearly distinguishable from those which are not. For each spot, six fields of view of equal size covering the full circumference were photographically recorded and the number of cells in each field were added. The number of cells can be either counted manually, or determined using image analysis software, ImageJ/Fiji^69^. We found no significant difference between the results between manual counting compared with that obtained by ImageJ. The values reported herein are the average of at least three independent experiments and the error bars represent standard deviation.

For tracking of cells and analysis of chemotaxis we respectively used ‘Manual Tracking’ and ‘Chemotaxis and Migration Tool’ plugins available through ImageJ/Fiji^69^. Chemotaxis parameters were obtained directly from this software and are defined as follows. The center of mass represents the averaged point of all cell endpoints and its x and y values indicate the direction in which the group of cells primarily travelled.

Graph construction and statistical analysis was performed using GraphPad Prism 6. Differences among groups were assessed using the ANOVA test. Differences between two groups were assessed using a *t* test. A P value less than 0.05 was considered statistically significant.

## Electronic supplementary material

Supplementary Information S1A

Supplementary Information S1B

Supplementary Information S1C

Supplementary Information S2

Supplementary Information S3-S5
