# Supplementary material for: Agarose Spot as a Comparative Method for in situ Analysis of Simultaneous Chemotactic Responses to Multiple Chemokines
Source: Sci Rep. 2017 Apr 21;7:1075. doi: 10.1038/s41598-017-00949-4 (PMC5430824; doi:10.1038/s41598-017-00949-4)
Supplement: Supplementary file 5 — Supplementary Information S3-S5 [file 41598_2017_949_MOESM5_ESM.pdf]

Supplementary Information

Agarose Spot as a Comparative Method for *in situ* Analysis of Simultaneous Chemotactic Responses to Multiple Chemokines

*Mohaned Ahmed,[1] Haneen A. Basheer,[1] Jose M. Ayuso,[2$] Djevdet Ahmet,[1] Marco Mazzini,[3] Roshan Patel,[1] Steven D. Shnyder,[1] Victoria Vinader,[1] Kamyar Afarinkia[1]**

[1] *The Institute of Cancer Therapeutics, University of Bradford, West Yorkshire BD7 1DP, United Kingdom.*
[2] *Group of Structural Mechanics and Material Modelling, Universidad Zaragoza, Zaragoza, Spain.*
[3] *Dipartimento di Scienza e Tecnologia del Farmaco, Universitá Degli Studi di Torino, Via P. Giuria 9, 10125, Torino, Italy.*

[$] *Current address: Department of Biomedical Engineering, Wisconsin Institutes for Medical Research, and The University of Wisconsin Carbone Cancer Center Madison, University of Wisconsin-Madison, Madison, WI 53715, USA.*

Correspondence should be addressed to (k.afarinkia@bradford.ac.uk)

Supplementary Information S1. (**A**) Video showing movement of PC-3 cells under an agarose spot containing CCL21. (**B**) Video showing tracking of PC-3 cells movements adjacent to an agarose spot containing CCL21. (**C**) Video showing tracking of PC-3 cells movements under an agarose spot containing CCL21.

Supplementary Information S2. A video tutorial for agarose spot assay

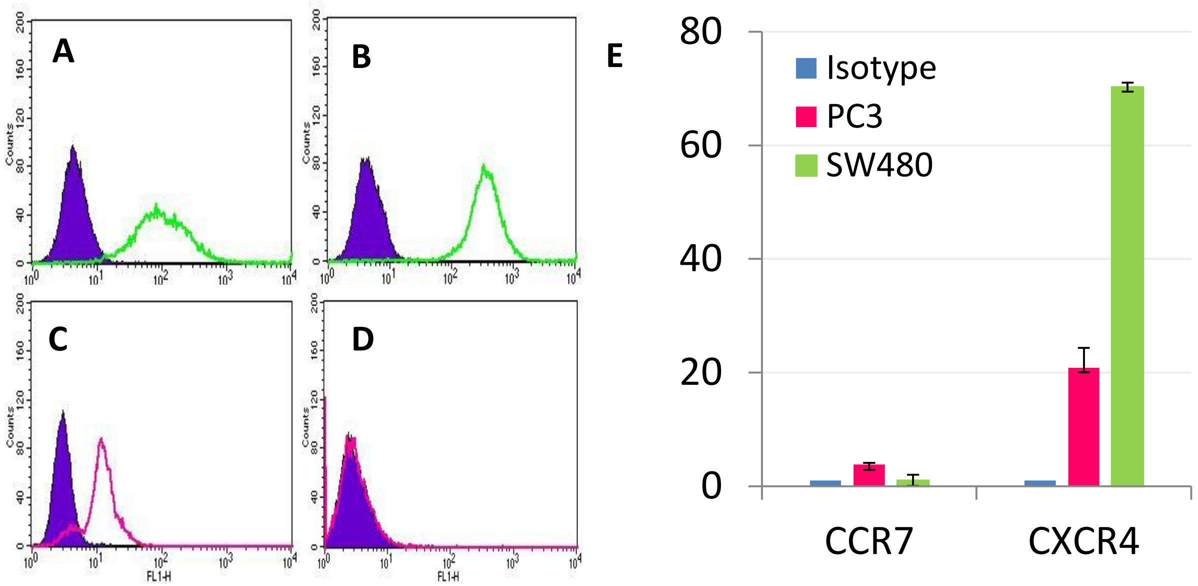

Supplementary Information S3. Expression of CXCR4 (green) compared with isotype (filled purple) in: (**A**) PC-3 cells; (**B**) SW480 cells; Expression CCR7 (cyan) compared with isotype (filled purple) in: (**C**) PC-3 cells; (**D**) SW480 cells; (**E**)Relative expression of CCR7 and CXCR4 relative to isotype control.

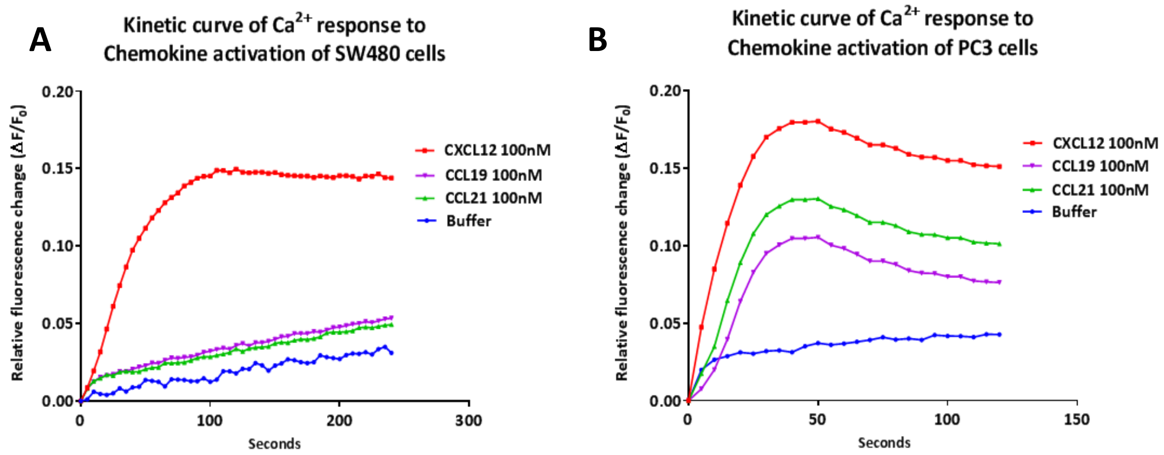

Supplementary Information S4. (**A**) Calcium mobilisation (Ca^{2+} flux) assay shows SW480 cells respond to 100 nM CXCL12 (red), but not to CCL21 (green), and CCL19 (purple). (**B**) calcium mobilisation (Ca^{2+} flux) assay shows PC-3 cells respond to 100 nM CXCL12 (red), CCL21 (green), and CCL19 (purple). Control (assay buffer, blue) shown for comparison.

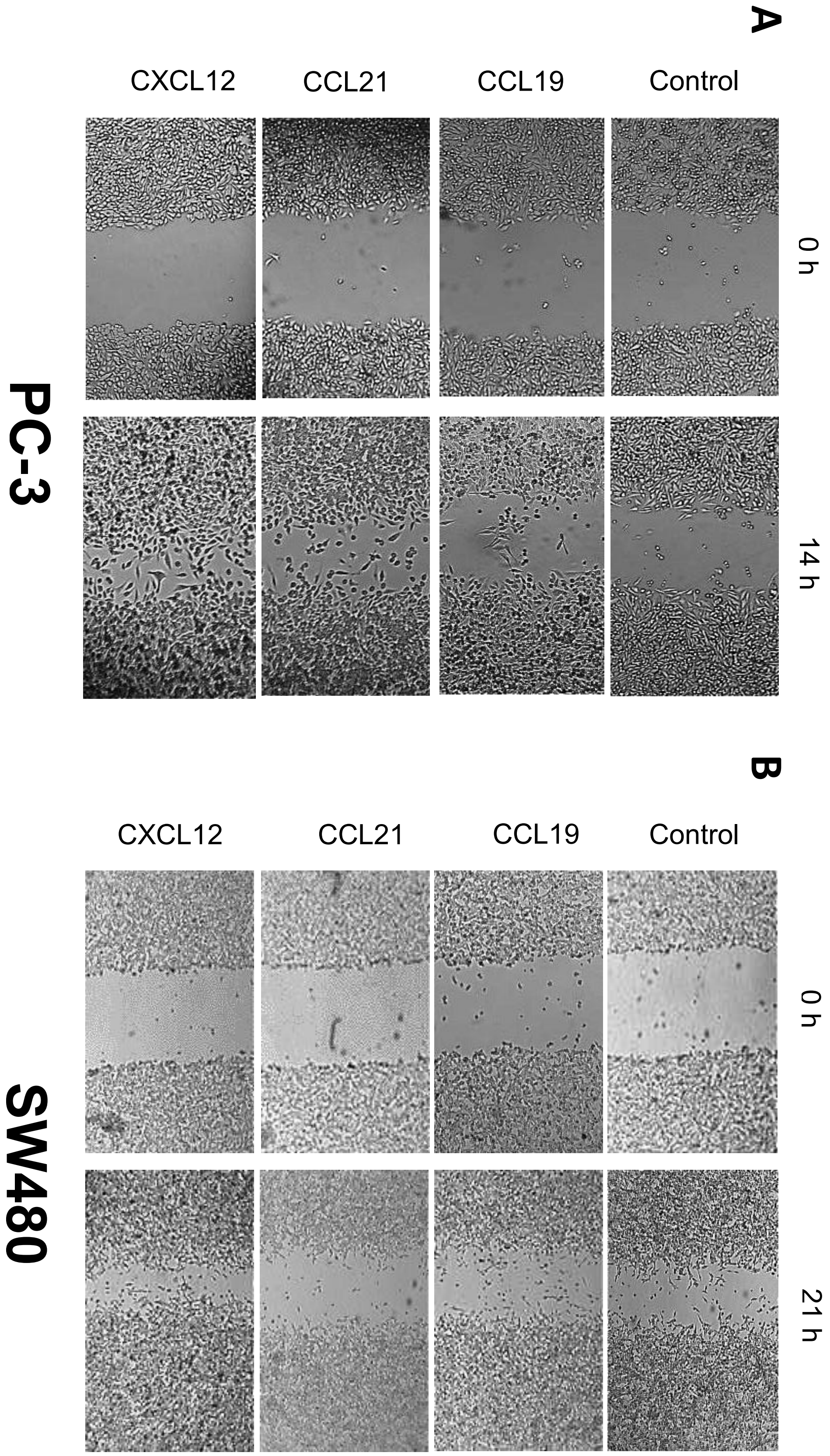

Supplementary Information S5. (**A**) Scratch assay showing the responses to CXCL12, CCL21, CCL19 (10nM) and control for PC-3 cells. (**B**) Scratch assay showing the responses to CXCL12, CCL21, CCL19 (10nM) and control for SW480 cells.
